# Digital Device-Related Interference, Living Alone, and Unmet Healthcare Needs: A Population-Based Study of Healthcare Access Barriers

**DOI:** 10.3390/healthcare14162594

**Published:** 2026-08-18

**Authors:** Hyerang Kim, Joohyun Chung, Wonyoung Cho, Heesook Son

**Affiliations:** 1 Department of Nursing Science, Howon University, 64 Howondea 3-gil, Impi-myeon, Gunsan-si 54508, Jeonbuk, Republic of Korea; hkim167@hotmail.com; 2 College of Nursing, University of Massachusetts Amherst, 224 Skinner Hall, Amherst, MA 01003, USA; joohyunchung@umass.edu; 3 Red Cross College of Nursing, Chung-Ang University, 84 Heukseok-ro, Dongjak-gu, Seoul 06974, Republic of Korea; elizabetcho@naver.com

**Keywords:** unmet healthcare needs, health services accessibility, digital technology, living alone, healthcare barriers

## Abstract

**Background/Objectives:** To examine the associations between digital device-related interference and living alone with unmet healthcare needs and barrier types in a nationally representative sample of Korean adults. This study had a cross-sectional design. **Methods:** Data from the 2023 Korea Community Health Survey (n = 213,820) were analyzed. Weighted multivariable logistic regression analyses were used to examine the association between digital device-related interference and living alone with unmet healthcare needs. Among those with unmet healthcare needs, additional analyses examined the associations with reporting individual-level rather than external barriers. **Results:** Overall, 5.7% of adults reported unmet healthcare needs. Digital device-related interference was associated with higher odds of unmet healthcare needs (adjusted odds ratio [aOR] = 1.696, 95% confidence interval [CI]: 1.569–1.834), whereas digital non-use was not. Living alone was also associated with higher odds of unmet healthcare needs (aOR = 1.277, 95% CI: 1.171–1.392). Among those with unmet healthcare needs, digital interference was associated with higher odds of reporting individual-level barriers, whereas living alone was associated with lower odds. No significant interactions were observed. **Conclusions:** These findings suggest that unmet healthcare needs in highly digitized societies may arise through distinct behavioral and structural pathways. Therefore, public health strategies to improve healthcare access should address not only the structural barriers faced by individuals living alone but also functional disruptions related to digital device use. These findings should be interpreted in light of cross-sectional design and reliance on self-reported data.

## 1. Introduction

Unmet healthcare needs occur when individuals require medical care but do not receive appropriate services and are widely used as indicators of health system performance and health inequities [1,2]. Beyond a simple measure of access, unmet healthcare needs reflect barriers arising at multiple stages of healthcare utilization, including financial, logistical, and time-related constraints [3]. Previous research has identified socioeconomic, health-related, and structural determinants of unmet healthcare needs, and examining the underlying barriers may provide more actionable evidence for public health planning [2,3]. Living arrangements have also been recognized as important social determinants of health. Individuals living alone may experience reduced social support and greater difficulties in navigating healthcare services [4]. Limited social support and social isolation have likewise been associated with higher levels of unmet healthcare needs [5,6].

Digital technology is becoming increasingly central to healthcare navigation and information-seeking in contemporary healthcare systems. Prior research has primarily emphasized digital access, suggesting that individuals who do not use digital devices may face disadvantages in obtaining health information and services [7]. However, healthcare-related digital engagement extends beyond simple access to digital devices and involves how individuals seek, understand, evaluate, and use digital health information and digitally mediated healthcare services. Differences in these abilities and patterns of engagement may contribute to digital inequalities in healthcare use, even in settings where access to digital devices is widespread. Within this broader context, vulnerabilities in highly digitized societies may arise not only from limited access or digital health literacy but also from the functional consequences of digital engagement. Despite growing interest in the functional consequences of digital engagement, limited population-based research has examined whether digital device-related interference is associated with unmet healthcare needs or the types of barriers individuals report. Digital device-related interference has been discussed in the literature on problematic mobile phone use; it has been characterized by disruptions to sleep, concentration, and daily routines, as a pattern of dysregulated digital engagement that may interfere with daily functioning [8]. Such disruptions may be associated with difficulties in time management, attention, and self-regulation, which could, in turn, hinder an individual’s ability to recognize health needs, prioritize health concerns, schedule medical appointments, and respond promptly to emerging symptoms. Consequently, digital interference may be associated with delayed or forgone care, although the direction of this relationship remains uncertain.

Living alone may also shape healthcare access by limiting instrumental or informational support within the household [4]. Given the global increase in single-person households [9] and continued expansion of digital device use [10], digital device-related interference and living alone may represent distinct yet complementary correlates of unmet healthcare needs. However, few population-based studies have jointly examined digital device-related interference, living arrangements, and the types of barriers underlying unmet healthcare needs. Therefore, in this study, we aimed to examine the association between digital device-related interference and living alone with unmet healthcare needs and barrier types in a nationally representative sample of Korean adults. Specifically, we sought to examine whether digital device-related interference was associated with unmet healthcare needs, including the likelihood of reporting individual-level rather than external barriers among those with unmet healthcare needs, and to assess whether living alone was independently associated with unmet healthcare needs within the same analytical framework.

## 2. Materials and Methods

### 2.1. Study Design

This was a cross-sectional secondary analysis of data from the 2023 Korea Community Health Survey.

### 2.2. Study Population and Sampling

The Korea Community Health Survey is an annual nationwide survey conducted by the Korea Disease Control and Prevention Agency to monitor health status and behaviors among community-dwelling Korean adults aged 19 years and older [11]. The survey employed a multistage stratified cluster sampling design to obtain a nationally representative sample of the Korean population. Trained interviewers conducted standardized face-to-face interviews using a structured questionnaire. All respondents with complete information on unmet healthcare needs, digital device-related interference, and living arrangements were included in the analysis. Sampling weights, stratification variables, and primary sampling units were applied to account for the complex survey design and generate nationally representative estimates. The final sample comprised 213,820 adults.

### 2.3. Measures

Unmet healthcare needs were assessed using a binary variable based on the question, “During the past 12 months, was there any time when you needed medical care but did not receive it?” Responses were coded as yes or no. Participants with missing responses were excluded from the analysis of this outcome.

Among participants reporting unmet healthcare needs, the reasons were categorized into two broad groups: external and individual-level barriers. External barriers refer to system-level or environmental constraints, including financial burdens, limited appointment availability, long waiting times, transportation difficulties, work or caregiving responsibilities, and pandemic-related service disruptions. Individual-level barriers refer to personal perceptions or circumstances, such as perceiving symptoms as mild, expecting spontaneous recovery, or fear of medical procedures. Responses categorized as “other” were treated as missing.

Digital device-related interference was assessed using a self-reported item with four response categories: never used, used with no interference, some interference, and interference almost daily. As this study focused on the presence of disruptive digital use rather than its severity, the categories “some interference” and “interference almost every day” were combined. This decision also enhanced the stability of the estimate because of the small number of respondents in the highest category. Therefore, digital interference was categorized into three groups for analysis: never used, used with no interference, and any interference. This item was selected because it was the only available Korea Community Health Survey item that directly assessed perceived interference of digital device use with daily functioning. As a single-item measure, internal consistency reliability could not be evaluated, and separate psychometric validation data were not available.

Living arrangements were defined as a dichotomous variable indicating whether the participant lived alone (yes or no). Covariates were selected based on the prior literature on healthcare access and included age, sex, educational attainment, employment status, marital status, household income, residential area, and subjective health status.

### 2.4. Statistical Analysis

Descriptive statistics were calculated to summarize the distribution of the study variables. In addition to *p*-values, standardized mean differences (SMDs) were calculated to assess the magnitude of the differences in general characteristics between the groups. Given the large sample size, SMDs were used to evaluate practical significance, independent of statistical significance. An SMD of less than 0.10 was considered negligible, 0.10–0.20 as small, and greater than 0.20 as meaningful. Multivariable logistic regression analyses were conducted to examine the association between digital device-related interference and unmet healthcare needs (yes/no). Among participants who reported unmet healthcare needs, additional logistic regression analyses were performed to examine the association between reporting individual-level and external barriers.

All analyses accounted for the complex survey design by incorporating sampling weights, stratification variables, and primary sampling units. Digital interference was entered as a three-level categorical variable (never used, used with no interference, and any interference), with “used with no interference” as the reference category. Living arrangements (living alone: yes/no) were included as an independent variable, and the interaction terms between digital interference and living arrangements were tested. All models were adjusted for age, sex, educational attainment, employment status, marital status, household income, residential area, and subjective health status. Adjusted odds ratios (aORs) and 95% confidence intervals (CIs) were estimated. Statistical significance was set at *p* < 0.05. Analyses were performed using SPSS version 29.0 (IBM Corp., Armonk, NY, USA).

## 3. Results

Table 1 presents the weighted characteristics of the study population according to unmet healthcare needs. Overall, 5.7% of the participants reported unmet healthcare needs. Although several variables showed statistically significant differences, most had negligible effect sizes (SMD < 0.10), indicating limited practical differences between groups. The largest difference was observed for subjective health status (SMD = 0.308). Meaningful differences were also identified for digital device-related interference (18.6% vs. 11.2%, SMD = 0.209) and living alone (19.2% vs. 14.1%, SMD = 0.137), whereas digital non-use showed a negligible difference (SMD = 0.013). Among participants with unmet healthcare needs, external barriers were more common than individual-level barriers (69.2% vs. 30.8%).

Table 2 shows the adjusted associations with unmet healthcare needs. Living alone and any digital device-related interference were each independently associated with higher odds of unmet healthcare needs (aOR = 1.277, 95% CI: 1.171–1.392; aOR = 1.696, 95% CI: 1.569–1.834, respectively). Digital non-use was not significantly associated with unmet healthcare needs. No significant interaction was observed between living alone and digital interference, indicating that these associations did not differ according to living arrangements.

Among participants with unmet healthcare needs (n = 12,153), Table 3 presents the adjusted associations with reporting individual-level barriers, rather than external barriers. Living alone was associated with lower odds of reporting individual-level barriers (aOR = 0.794, 95% CI: 0.675–0.934), indicating a greater likelihood of reporting external barriers among those living alone. In contrast, any digital device-related interference was associated with higher odds of reporting individual-level barriers (aOR = 1.290, 95% CI: 1.118–1.488), whereas digital non-use was associated with lower odds. No significant interaction was observed between living arrangements and digital interference. Older age, urban residence, and better subjective health were associated with higher odds of reporting individual-level barriers, whereas employment was associated with lower odds.

## 4. Discussion

The findings of this study suggest that unmet healthcare needs in highly digitized societies may arise from distinct domains of vulnerability. Digital device-related interference was associated with higher odds of unmet healthcare needs and a greater likelihood of reporting individual-level barriers, whereas living alone was independently associated with unmet healthcare needs and a lower likelihood of reporting individual-level barriers, indicating a greater relative contribution of external barriers. No significant interaction was observed between digital device-related interference and living arrangements. Collectively, these findings suggest that unmet healthcare needs may reflect parallel behavioral and structural pathways rather than a single, uniform pattern of disadvantage.

A key finding of this study was that digital device-related interference, rather than digital non-use, was associated with unmet healthcare needs. Early digital divide research primarily focused on inequalities in access to digital technologies and Internet connectivity [12]. However, in highly digitized societies with widespread access to digital devices, disparities in healthcare access may arise less from whether individuals use digital devices and more from how digital engagement affects daily functioning. Disruptive digital engagement may be associated with difficulties in time management, attention, and self-regulation, which could, in turn, be related to delays in recognizing health needs or seeking timely care; however, the cross-sectional design does not allow the direction of these associations to be determined. Prior research has suggested that procrastination and self-regulatory difficulties may reduce engagement in preventive health behaviors, such as cancer screening and cholesterol testing [13]. The present findings extend this perspective by suggesting that functional interference related to digital device use may also be relevant to unmet healthcare needs. However, this proposed behavioral pathway remains hypothetical because the present dataset did not include direct measures of self-regulation, executive functioning, health literacy, or healthcare navigation skills. Therefore, the observed association should not be interpreted as evidence that these mechanisms explain the relationship between digital device-related interference and unmet healthcare needs.

The association between digital device-related interference and individual-level barriers further suggests that unmet healthcare needs in this group may be linked less to structural access limitations and more to difficulties in prioritizing or acting on health concerns. This pattern is consistent with the possibility that disruptive digital engagement may affect self-regulation and day-to-day organization in ways that delay healthcare-seeking. Notably, SMDs also indicated meaningful group differences for digital device-related interference, whereas most other sociodemographic variables showed negligible effect sizes despite their statistical significance. Collectively, these findings support the practical relevance of digital device-related interference as a contextual factor associated with unmet healthcare needs. Therefore, public health strategies may benefit from identifying whether vulnerability is more closely related to behavioral or structural barriers and aligning interventions accordingly.

In contrast to digital device-related interference, living alone was more strongly associated with external barriers to healthcare. This pattern suggests that unmet healthcare needs among individuals living alone may be shaped primarily by structural and logistical constraints rather than by individual-level perceptions or behavioral factors. Individuals living alone may have less access to instrumental or informational support within the household, which may limit their ability to coordinate care, arrange transportation, and navigate healthcare services [14]. Therefore, the implications of household structure for healthcare access may become increasingly important. in aging and urbanizing societies.

Although no significant interaction was observed between digital device-related interference and living arrangements, this finding remains informative. One possible explanation is that living alone and digital device-related interference may operate through different mechanisms, with the former reflecting structural and support-related constraints and the latter reflecting individual behavioral or functional difficulties. In addition, living alone does not necessarily imply social isolation, and digital device-related interference does not necessarily indicate problematic digital engagement; therefore, the overlap between the two vulnerabilities may have been weaker than theoretically expected. Rather than synergistically reinforcing each other, these factors may represent independent domains of vulnerability associated with unmet healthcare needs. This interpretation is consistent with the observed pattern in which digital device-related interference was more closely associated with individual-level barriers, whereas living alone was more strongly linked to external barriers. Therefore, efforts to reduce unmet healthcare needs may require different strategies depending on whether barriers are primarily behavioral or structural.

Another finding was that digital non-use was not significantly associated with unmet healthcare needs. This suggests that digital exclusion may not uniformly translate into healthcare access disadvantages in contexts where offline pathways to care remain available. Although lack of access has often been conceptualized as a central dimension of digital inequality [12], the present findings suggest that in highly digitized but still mixed delivery systems, non-use alone may not be sufficient to produce unmet healthcare needs. Instead, disparities may increasingly relate to how digital engagement affects daily functioning rather than whether digital devices are used at all.

Unmet healthcare needs were also more likely among employed individuals and those with relatively higher incomes. This pattern suggests that unmet healthcare needs are not limited to socioeconomically disadvantaged groups. In some contexts, unmet healthcare needs may reflect time-related constraints rather than financial barriers. Competing work demands, rigid schedules, and limited flexibility may reduce the ability to seek timely care despite adequate financial resources, given that time-related constraints have been identified as important non-financial barriers to healthcare access [15]. These findings underscore the importance of considering temporal barriers alongside economic factors when examining inequalities in healthcare access.

Among individuals reporting unmet healthcare needs, external barriers accounted for most cases. This predominance underscores the continuing importance of structural conditions in shaping access to care. Even in highly digitized societies, financial burden, transportation difficulties, service availability, and other system-level constraints remain important determinants of unmet healthcare needs [1]. These findings caution against overemphasizing individual responsibility in healthcare-seeking and reinforce the need to address persistent structural barriers within healthcare systems.

This study has certain limitations. First, its cross-sectional design precludes causal inferences and does not allow the temporal ordering of digital device-related interference and unmet healthcare needs to be determined. Although digital interference may plausibly be related to difficulties in attention, time management, or daily routines, reverse causation is also possible. For example, individuals experiencing health concerns or difficulty accessing care may engage more extensively with digital devices or perceive greater interference in their daily lives. Therefore, the observed association should not be interpreted as evidence that digital interference causes unmet healthcare needs. Longitudinal studies with repeated assessments of digital use and healthcare utilization are needed to establish temporality and evaluate potential causal pathways. Second, all measures were based on self-report and may have been subject to recall bias or subjective interpretation. Participants may not have accurately recalled unmet healthcare needs or the extent to which digital device use interfered with their daily lives over the previous 12 months, potentially resulting in exposure or outcome misclassification. Such measurement error is likely to be non-differential, which would generally bias the observed associations toward the null rather than inflate them. In addition, digital interference was assessed using a single self-reported item rather than a validated multidimensional instrument, which may have limited construct validity and increased the possibility of measurement error. The item did not capture more detailed behavioral patterns, including the frequency, severity, context, or specific functional consequences of digital device-related interference, or objective usage data. Future studies should incorporate multidimensional measures that assess the frequency, duration, and context of digital device use, as well as specific functional consequences, such as interference with sleep, concentration, daily routines, and healthcare-seeking behaviors. The inclusion of objective indicators, such as device-recorded screen time or application-use data, may further improve measurement precision and facilitate the examination of potential dose–response relationships. Third, although digital interference was categorized to reflect the presence of disruptive use rather than its severity, this approach may have obscured potential dose–response relationships. Finally, despite the inclusion of a broad range of sociodemographic covariates, residual confounding by unmeasured factors remains possible. Although information on mental health was available in the survey, it was not included in the present analyses because the primary objective was to examine the association between digital device-related interference and unmet healthcare needs while adjusting for key sociodemographic characteristics. In addition, health literacy and more detailed indicators of social support were not available in the Korea Community Health Survey dataset. Digital device-related interference may also reflect underlying psychological conditions, such as anxiety, depression, or stress, which may independently influence healthcare-seeking behaviors and unmet healthcare needs. Because these factors may be associated with both digital device-related interference and unmet healthcare needs, residual confounding by psychological factors cannot be excluded. Future longitudinal studies incorporating these variables are warranted to better understand the mechanisms underlying unmet healthcare needs.

Despite these limitations, this study has several strengths. It used nationally representative data with complex survey weighting, enhancing the generalizability of the findings. It also extends prior research on digital inequality by conceptualizing digital vulnerability not only in terms of access but also in terms of functional interference in daily life. Furthermore, by distinguishing between external and individual-level barriers among those reporting unmet healthcare needs, this study provides more nuanced insights into the mechanisms underlying healthcare access disparities. The simultaneous examination of digital device-related interference and living arrangements further supports the interpretation that unmet healthcare needs in highly digitized settings may arise through independent behavioral and structural pathways.

## 5. Conclusions

Digital device-related interference and living alone were independently associated with unmet healthcare needs, although no significant interaction was observed. Digital interference was more closely linked to individual-level barriers, whereas living alone was more strongly associated with external barriers. These findings suggest that unmet healthcare needs in highly digitized societies may arise through distinct behavioral and structural pathways. Therefore, public health efforts to reduce unmet healthcare needs may benefit from barrier-specific strategies that address both disruptive digital engagement and the structural challenges faced by individuals living alone.

## Figures and Tables

**Table 1:** Weighted Descriptive Characteristics of the Study Population According to Unmet Healthcare Needs.

Variable	Total (n = 213,820)	No Unmet Needs (n = 201,667)	Unmet Needs (n = 12,153)	*p*-Value	SMD ^a^
Age (mean ± SE)	50.75 ± 0.06	50.85 ± 0.06	48.95 ± 0.20	<0.001	0.110
Sex					
Male	95,845 (48.6)	91,085 (48.9)	4760 (43.7)	<0.001	0.104
Female	117,975 (51.4)	110,582 (51.1)	7393 (56.3)		
Education					
Less than high school	68,998 (18.7)	64,867 (18.7)	4131 (19.3)	<0.001	0.015
High school Graduate	61,697 (28.6)	58,179 (28.5)	3518 (30.2)		0.037
College or higher	83,030 (52.7)	78,536 (52.9)	4494 (50.5)		0.048
Employment					
Employee	38,578 (14.7)	36,229 (14.6)	2349 (16.6)	<0.001	0.055
Employer	88,705 (48.5)	83,638 (48.5)	5067 (48.4)		0.002
Not employed/other	86,513 (36.8)	81,777 (36.9)	4736 (34.9)		0.042
Marital status					
Married	136,322 (61.9)	129,435 (62.2)	6887 (55.4)	<0.001	0.138
Separated/divorced/widowed/never married	77,459 (38.1)	72,199 (37.8)	5261 (44.6)		
Household income					
Lower than median	103,238 (61.0)	97,830 (61.2)	5408 (56.3)	<0.001	0.100
Higher than median	107,566 (39.0)	100,980 (38.8)	6586 (43.7)		
Residential area					
Urban	120,419 (81.3)	113,845 (81.4)	6574 (79.7)	<0.001	0.043
Rural	93,401 (18.7)	87,822 (18.6)	5579 (20.3)		
Subjective health status					
Good	78,085 (42.1)	75,119 (43.0)	2966 (28.4)	<0.001	0.308
Fair	92,183 (43.4)	86,967 (43.2)	5216 (46.9)		0.074
Poor	43,550 (14.5)	39,579 (13.8)	3971 (24.8)		0.281
Living alone	38,837 (14.4)	35,964 (14.1)	2873 (19.2)	<0.001	0.137
Digital interference					
Never use (yes)	39,145 (10.7)	36,730 (10.7)	2415 (11.1)	<0.001	0.013
No interference (yes)	157,368 (77.6)	149,251 (78.1)	8117 (70.3)		0.179
Any interference (yes)	17,282 (11.7)	15,666 (11.2)	1616 (18.6)		0.209
Barrier type					
External barriers		-	8212 (69.2)		
Individual-level barriers		-	3341 (30.8)		

^a^ SMD: Standardized Mean Difference.

**Table 2:** Adjusted Odds Ratios for Unmet Healthcare Needs.

Variable	aOR ^a^	95% CI ^b^	*p*-Value
Age (year)	0.984	0.981–0.986	<0.001
Sex (ref: Female)			
Male	0.785	0.748–0.825	<0.001
Education (ref: College or higher)			
Less than high school	1.142	1.045–1.248	0.003
High school graduate	1.191	1.118–1.268	<0.001
Employment (ref: Not employed/Other)			
Employer/Self-employed	1.543	1.433–1.661	<0.001
Employee/Wage worker	1.217	1.147–1.292	<0.001
Marital status (ref: Separated/Divorced/Widowed/Never married)			
Married	0.996	0.933–1.063	0.896
Household income (ref: Higher than median)			
Lower than median	0.868	0.817–0.923	<0.001
Residential area (ref: Rural)			
Urban	0.927	0.876–0.980	0.008
Subjective health (ref: Poor)			
Good	0.287	0.267–0.309	<0.001
Fair	0.508	0.478–0.540	<0.001
Living Alone (yes)	1.277	1.171–1.392	<0.001
Digital interference			
Never use (yes)	1.085	0.995–1.183	0.066
Any interference (yes)	1.696	1.569–1.834	<0.001
Interaction			
Living alone × never use	1.010	0.870–1.174	0.891
Living alone × any interference	0.882	0.744–1.047	0.151

^a^ aOR = adjusted odds ratio, ^b^ CI = confidence interval.

**Table 3:** Factors associated with individual-level barriers among adults reporting unmet healthcare needs.

Variable	aOR ^a^	95% CI ^b^	*p*-Value
Age (year)	1.013	1.010–1.017	<0.001
Sex (ref: Female)			
Male	1.086	0.986–1.197	0.094
Education (ref: College or higher)			
Less than high school	0.899	0.760–1.063	0.213
High school graduate	1.120	0.993–1.264	0.065
Employment (ref: Not employed/Other)			
Employer/Self-employed	0.484	0.419–0.559	<0.001
Employee/Wage worker	0.539	0.480–0.605	<0.001
Marital status (ref: Separated/Divorced/Widowed/Never married)			
Married	1.042	0.941–1.154	0.431
Household income (ref: Higher than median)			
Lower than median	1.063	0.946–1.194	0.305
Residential area (ref: Rural)			
Urban	1.353	1.217–1.504	<0.001
Subjective health (ref: Poor)			
Good	2.993	2.584–3.465	<0.001
Fair	2.396	2.094–2.741	<0.001

^a^ aOR = adjusted odds ratio, ^b^ CI = confidence interval.

## Data Availability

The data used in this study are publicly available from the Korea Community Health Survey (KCHS), conducted by the Korea Disease Control and Prevention Agency. Information on data access is available through the KCHS website after completion of the required data use application procedures.

## Abbreviations

The following abbreviations are used in this manuscript:

CI	Confidence interval
KCHS	Korea Community Health Survey
SMD	Standardized mean differences
